# Recuperação da flexão do cotovelo e função respiratória em pacientes com lesão traumática total do plexo braquial tratados com transferência do nervo frênico

**DOI:** 10.1055/s-0045-1811931

**Published:** 2025-11-21

**Authors:** Giovanni V.C. Guedes, Rogério R. Visconti, Rudolf N. Kobig, João A. M. Guimarães, Conrado T. Laett

**Affiliations:** 1 Laboratório de Pesquisa Neuromuscular, Divisão de Ensino e Pesquisa, Instituto Nacional de Traumatologia e Ortopedia Jamil Haddad, Rio de Janeiro, RJ, Brasil

**Keywords:** nervo frênico, plasticidade neuronal, plexo braquial/lesões, transferência de nervo, brachial plexus/injuries, nerve transfer, neuronal plasticity, phrenic nerve

## Abstract

**Objetivo:**

Avaliar os resultados da transferência do nervo frênico em pacientes com lesão traumática total do plexo braquial, especialmente a flexão do cotovelo e a função respiratória.

**Métodos:**

Trata-se de uma série de casos de 16 pacientes submetidos à transferência do nervo frênico entre 2014 e 2021. Foram incluídos pacientes com mais de 18 anos, operados havia mais de 6 meses, e sem outras doenças ortopédicas do membro superior. A força de flexão do cotovelo foi avaliada pela escala do Medical Research Council (MRC), por dinamometria isocinética e por eletromiografia. A função respiratória foi analisada por espirometria.

**Resultados:**

Os pacientes eram principalmente homens jovens que sofreram acidentes com motocicletas. Na escala do MRC, 37,5% dos pacientes atingiram o nível III, e 43,8%, o nível IV. Em média, a força de flexão do cotovelo foi de 9,1% em comparação à do braço não acometido. O estudo identificou déficits inconsistentes na função respiratória, sem comprometimento grave da capacidade vital forçada e do volume expiratório forçado. Não houve relatos de sintomas respiratórios. A ativação involuntária do bíceps braquial foi observada durante ciclos respiratórios forçados, com pico após um período inicial de recuperação.

**Conclusão:**

A transferência do nervo frênico foi eficaz na recuperação da flexão do cotovelo na maioria dos pacientes. Observamos sinais de neuroplasticidade que melhoraram o controle motor do braço ao longo do tempo. Os pacientes não apresentaram evidências de comprometimento pulmonar grave.

## Abstract (en)

**Objective:**

To evaluate the outcomes of phrenic nerve transfer in total traumatic brachial plexus injury, focusing on elbow flexion and respiratory function.

**Methods:**

The present is a case series of 16 patients undergoing phrenic nerve transfer between 2014 and 2021. Patients over 18 years old, operated on for more than 6 months, and without other orthopedic conditions of the upper limb were included. Elbow flexion strength was assessed by Medical Research Council (MRC) scale and an isokinetic dynamometer, along with an electromyograph. Respiratory function was assessed by spirometry.

**Results:**

The patients were mainly young men affected by motorcycle accidents. Level III on the MRC was achieved by 37.5% of patients, with 43.8% reaching level IV. On average, elbow flexion strength was of 9.1% compared with that of the unaffected arm. The study identified inconsistent deficits in respiratory function, with no severe impairment in forced vital capacity and forced expiratory volume. Respiratory symptoms were not reported. Involuntary activation of the biceps brachii during forced respiratory cycles was observed, peaking after an initial recovery period.

**Conclusion:**

Phrenic nerve transfer effectively restored elbow flexion in most patients. We found signs of neuroplasticity that enhanced the motor control of the arm over time. We found no evidence of severe pulmonary impairment in these patients.

## Introdução

As lesões do plexo braquial prejudicam o controle dos membros superiores e a funcionalidade do paciente. A avulsão total do plexo braquial, que ocorre em 47% a 58% dos casos induzidos por trauma, é particularmente grave, e provoca perda completa do controle dos membros superiores. 1 Indivíduos com lesão traumática total do plexo braquial (LTTPB) não só sofrem perda de funcionalidade e autonomia, como também apresentam, com frequência, transtorno de estresse pós-traumático e ideação suicida. 2 Além disso, a LTTPB tem repercussões econômicas individuais e públicas. 3

Na ausência de resolução espontânea, há vários tratamentos cirúrgicos para tentar a recuperação parcial do controle do braço, desde neurólise até transferências nervosas. Nestes pacientes, a transferência de um nervo motor intacto para o controle de outros músculos é uma abordagem viável, bem descrita e capaz de restaurar o controle da flexão do cotovelo. 4 A transferência de Oberlin, do ramo motor do nervo ulnar para o ramo motor do bíceps braquial, destaca-se como um método eficaz para a recuperação da flexão do cotovelo, 5 e tornou-se a opção preferida em diversos serviços para o tratamento de lesões parciais.

Os casos de lesão total, porém, necessitam de um doador extraplexual devido ao comprometimento do nervo ulnar. Entre as opções estão os nervos frênico, espinhal acessório e intercostal. O nervo frênico tem sido considerado uma opção viável desde os primeiros relatos do cirurgião russo Lurje, 6 em 1948, e ainda é relevante, 4 pois há relatos encorajadores de recuperação da força de flexão do cotovelo. 7 8 9 No entanto, existem preocupações devido aos relatos de paralisia diafragmática associada, evidenciada por elevação da hemicúpula, e diminuição da força inspiratória apesar da ausência de sintomas respiratórios. 10 A capacidade vital forçada (CVF) pode ser um índice importante na avaliação desses pacientes, ao permitir a comparação da função respiratória em relação ao esperado, considerando a idade e o sexo do paciente. Valores acima de 80% do previsto são considerados normais, ao passo que valores de até 60% e 50% caracterizam distúrbios brandos e moderados, respectivamente, e valores abaixo de 50% indicam distúrbios graves. 11 Além das preocupações com a função respiratória, o controle motor do bíceps braquial reinervado pode ser difícil devido à função original distinta do nervo frênico, o que pode levar a contrações involuntárias durante o esforço respiratório.

Considerando as graves implicações da LTTPB, é crucial investigar tratamentos cirúrgicos que sejam eficazes e seguros. A transferência do nervo frênico parece ser uma opção viável para a recuperação de algum nível de flexão do cotovelo, mas seus impactos na função respiratória e no controle motor ainda são incertos. Assim, o objetivo deste estudo foi avaliar a produção de força de flexão do cotovelo e a função respiratória em pacientes com LTTPB submetidos à transferência do nervo frênico para o ramo motor do bíceps braquial.

## Materiais e Métodos

Esta série de casos prospectivos incluiu 16 pacientes submetidos à cirurgia no Serviço de Microcirurgia de nossa instituição entre 2014 e 2021. O projeto foi aprovado por um comitê independente de experimentação em seres humanos (CAAE: 50087221.5.0000.5273). Todos os pacientes assinaram o termo de consentimento livre e esclarecido antes da entrada no estudo. Os critérios de inclusão foram idade superior a 18 anos no momento da coleta de dados, período pós-operatório superior a 6 meses e ausência de outras doenças ortopédicas ou neurológicas no membro superior.

**Fig. 1:**
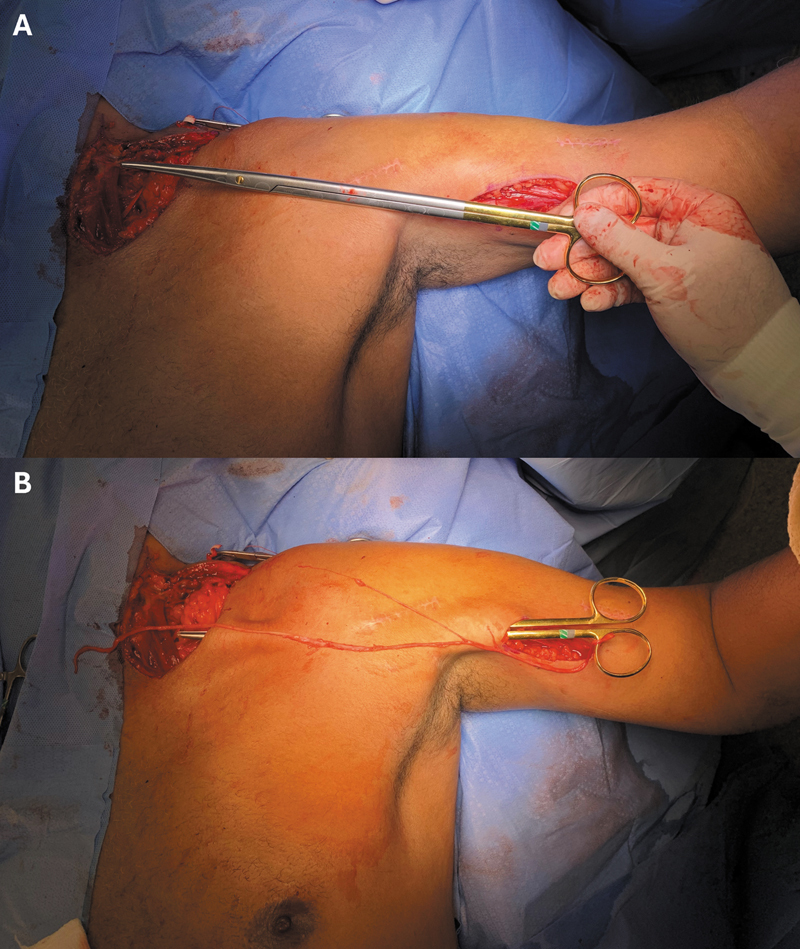
Planejamento ( **A** ) e construção ( **B** ) do túnel para a passagem do enxerto extraído do nervo sural utilizado na transferência do nervo frênico para o ramo motor do nervo musculocutâneo.

O procedimento cirúrgico começou com o paciente em decúbito dorsal para a retirada do enxerto do nervo sural do membro inferior contralateral. Utilizando uma abordagem supraclavicular, o nervo acessório foi transferido para o nervo supraescapular, e o nervo frênico foi dissecado. O ramo motor do nervo musculocutâneo foi acessado por uma abordagem medial no braço, e o enxerto foi passado para o nervo frênico por meio de um túnel preparado com uma tesoura romba ( Fig. 1 ). Suturas microcirúrgicas foram feitas proximal e distalmente ao enxerto, seguidas de fechamento da incisão e da colocação do membro superior em uma tipoia. A imobilização com tipoia foi mantida por 3 semanas. Em seguida, os pacientes foram encaminhados para o Departamento de Reabilitação para o controle da rigidez articular, a melhora da estabilidade postural e a promoção do controle motor do membro.

Os pacientes foram convidados a participar do projeto por telefone e e-mail. Todas as avaliações foram realizadas em uma só visita ao instituto. O questionário Disabilities of the Arm, Shoulder, and Hand (DASH) foi preenchido pelos pacientes, e a força de flexão do cotovelo foi avaliada subjetivamente por meio da escala do Medical Research Council (MRC) por um cirurgião de mão especializado em microcirurgia. A avaliação objetiva da força de flexão do cotovelo foi realizada por meio de contrações isométricas máximas a 90° de flexão do cotovelo utilizando um dinamômetro isocinético (Humac Norm III, Computer Sports Medicine, Inc.).

**Fig. 2:**
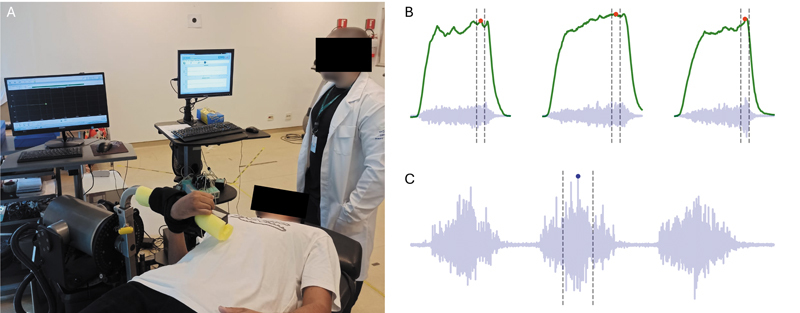
Avaliação da força muscular flexora do cotovelo e ativação do bíceps braquial ( **A** ). Sinal representativo do torque gerado na articulação do cotovelo (linha verde) e eletromiografia de superfície do bíceps braquial (linha azul) durante as três repetições do teste de força ( **B** ) e ciclos respiratórios forçados ( **C** ). As linhas verticais tracejadas indicam o período de 500 ms utilizado para o cálculo do valor da raiz quadrada média para a quantificação da ativação muscular.

Na avaliação com o dinamômetro, os pacientes foram posicionados em decúbito dorsal com o braço ao lado do tronco e a mão presa na alça do equipamento, de acordo com as instruções do fabricante ( Fig. 2A ). Após a familiarização com 3 contrações submáximas a 50% do esforço máximo, 3 contrações máximas foram realizadas em intervalos de 30 segundos. O valor máximo de torque em todas as contrações foi registrado para análise. Esse processo foi repetido enquanto os pacientes realizavam esforços inspiratórios e expiratórios máximos em ordem aleatória antes de cada contração máxima. O membro saudável foi testado primeiro para facilitar a compreensão e minimizar o desconforto. A ativação do bíceps braquial foi monitorada por eletromiografia (EMG) a 1 kHz (SAS1000; EMG System do Brasil) durante a contração máxima. A ativação do bíceps braquial também foi monitorada durante três ciclos respiratórios forçados. Em ambos os casos, o valor da raiz quadrada média em uma janela de 500 ms por volta do pico de EMG foi usado para quantificar a ativação do bíceps braquial. A ativação média ao longo das três contrações máximas foi usada para análise ( Fig. 2B ). A ativação do bíceps braquial durante os ciclos respiratórios forçados foi expressa como uma porcentagem da ativação observada durante a contração máxima ( Fig. 2C ).

**Fig. 3:**
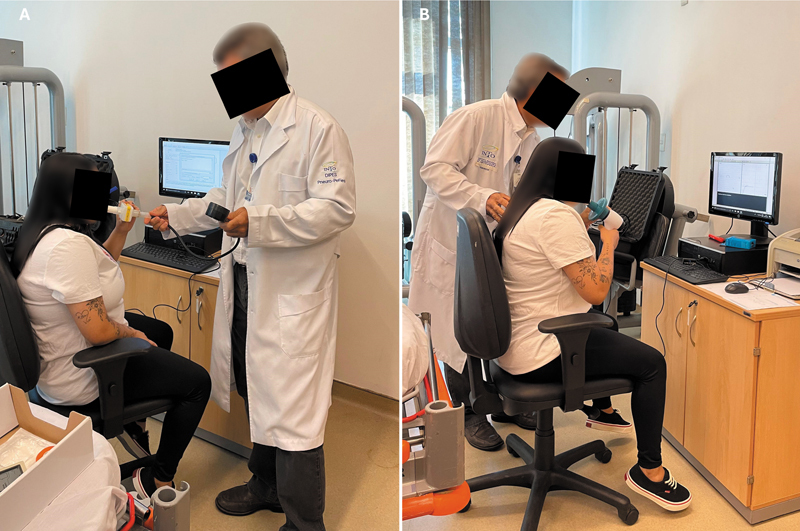
Avaliação da função respiratória conforme a capacidade vital forçada (CVF), o volume expiratório forçado no primeiro segundo (VEF1) e o índice de Tiffeneau-Pinelli (relação VEF1/CVF; **A** ), juntamente com as pressões inspiratória e expiratória máximas ( **B** ).

A função respiratória foi avaliada segundo a CVF, o volume expiratório forçado no primeiro segundo (VEF1) e o índice de Tiffeneau-Pinelli (relação VEF1/CVF), medidos com um pneumotacômetro tipo Fleisch (KoKo Sx1000, nSpireHealth) por um pneumologista experiente ( Fig. 3A ). Além disso, a pressão expiratória máxima (PEmáx) e a pressão inspiratória máxima (PImáx) foram medidas pelo mesmo profissional utilizando um manovacuômetro analógico (Wika) e expressas, assim como a CVF e o VEF1, como a porcentagem dos valores previstos para a população adulta brasileira 12 ( Fig. 3B ). A elevação da hemicúpula diafragmática, avaliada pela radiografia de tórax em plano frontal, foi definida como um aumento no ápice da estrutura em dois ou mais arcos costais acima da posição anatômica.

As variáveis numéricas apresentaram distribuição normal de acordo com o teste de Shapiro-Wilk, e foram expressas como valores de média ± desvio padrão. As diferenças na produção de força entre as manobras respiratórias foram testadas por análise de variância (ANOVA) unidirecional de medidas repetidas. Os déficits nas variáveis respiratórias foram verificados por testes *t* de uma amostra em relação ao valor de referência (100%). A tendência temporal da contração involuntária ao longo do tempo foi obtida por regressão polinomial de terceira ordem com o respectivo coeficiente de determinação (R ^2^ ). Todas as análises foram realizadas em rotinas personalizadas do Python 3.9 (grátis e de código aberto), e o nível α foi fixado em 0,05.

## Resultados

Dos 41 pacientes elegíveis, 27 (65,8%) puderam ser contatados, dos quais 16 (39%) concordaram em participar do estudo; suas características demográficas estão descritas na Tabela 1 . A função respiratória pareceu apresentar comprometimento parcial em comparação às estimativas em indivíduos saudáveis, embora as pressões respiratórias estivessem aparentemente preservadas ( Tabela 1 ). A elevação da hemicúpula diafragmática foi observada em 5 (37%) pacientes. Todos os pacientes sofreram lesões em acidentes automobilísticos, e quase todos, exceto um, pilotavam motocicletas. Ao acompanhamento, nenhum paciente relatou sintomas respiratórios.

**Tabela 1:** Características demográficas dos pacientes

**Idade média (anos)**	32 ± 10
**Homens: n (%)**	13 (81,2%)
**Altura média (m)**	1,68 ± 0,10
**Peso médio (kg)**	68,0 ± 14,1
**Tempo médio entre a lesão e a cirurgia (meses)**	7 ± 2
**Tempo médio de acompanhamento (meses)**	39 ± 28
**Força de flexão do cotovelo (escala do MRC): n (%)**	
** 1**	1 (6,2%)
** 2**	2 (12,5%)
** 3**	6 (37,5%)
** 4**	7 (43,8%)
**Força média de flexão do cotovelo (%)**	9,1 ± 7,0
**Pontuação média no DASH**	34 ± 11
**CVF média (%)**	81,1 ± 18,7*
**VEF1 médio (%)**	79,1 ± 17,6*
**Média do índice de Tiffeneau-Pinelli**	1,0 ± 0,1
**Média da PEmáx (%)**	103,9 ± 36,9
**Média da PImáx (%)**	108,1 ± 21,5

**Abreviaturas:** CVF, capacidade vital forçada; DASH, Disabilities of the Arm, Shoulder, and Hand; MRC, Medical Research Council; PEmáx, pressão expiratória máxima; PImáx, pressão inspiratória máxima; VEF1, volume expiratório forçado no primeiro segundo.

**Nota:** * Significativamente menor do que o estimado na população saudável, ou seja, 100% ( *p* < 0,05).

A força de flexão do cotovelo variou de 0,6% a 21,1%. Os 5 casos (31%) de recuperação acima de 10% ocorreram mais de 36 meses após a cirurgia. Ao todo, 7 participantes foram classificados como M4, 6, como M3, 2, como M2, e 1, como M1. Não observamos evidências de que manobras respiratórias antes da flexão do cotovelo influenciassem a produção de força (F2,24 = 1,04; *p* = 0,365).

**Fig. 4:**
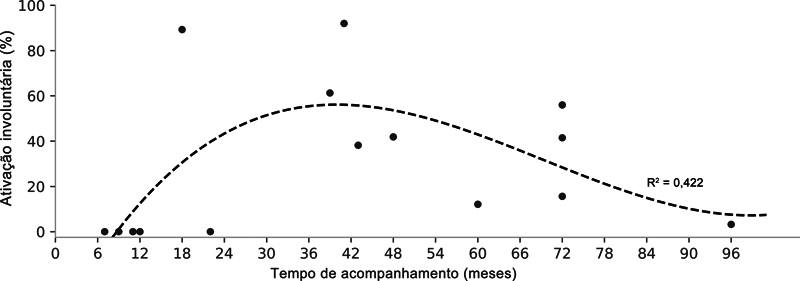
Contração involuntária do bíceps braquial durante ciclos respiratórios forçados no período de acompanhamento dos pacientes. Os pontos cinzas representam dados individuais, e a linha tracejada preta, a tendência derivada da regressão polinomial de terceira ordem, apresentada juntamente com o coeficiente de determinação (R ^2^ ).

Contrações involuntárias do bíceps braquial durante ciclos respiratórios forçados foram observadas em 11 pacientes, predominantemente nos primeiros 12 meses de acompanhamento. Posteriormente, foi identificado um nível de ativação de até 91,9%, seguido de uma tendência decrescente ao longo do tempo ( Fig. 4 ).

**Fig. 5:**
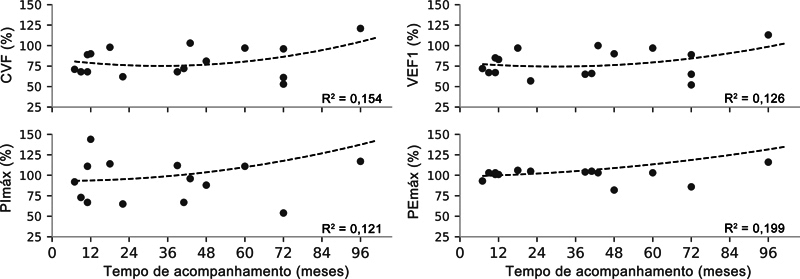
Função pulmonar longitudinal ao longo do acompanhamento dos pacientes. Os pontos cinzas representam dados individuais, e as linhas pretas tracejadas, a tendência derivada da regressão polinomial de terceira ordem, apresentada juntamente com o coeficiente de determinação (R ^2^ ). **Abreviaturas:** VEF1, volume expiratório forçado no primeiro segundo; CVF, capacidade vital forçada; PImáx, pressão inspiratória máxima; PEmáx, pressão expiratória máxima.

Valores normais de CVF foram observados em 8 pacientes (50%), ao passo que distúrbios restritivos brandos e moderados foram observados em 7 (43%) e 1 (6%) indivíduos, respectivamente. Não houve nenhum caso de distúrbio restritivo grave. Os parâmetros de função respiratória (CVF, VEF1, PImáx e PEmáx) apresentaram tendência temporal relativamente linear ( Fig. 5 ).

## Discussão

Nossos principais achados foram que a transferência do nervo frênico pode recuperar a flexão do cotovelo em diferentes níveis e que a extensão do comprometimento da função respiratória é incerta. Nossa amostra foi composta principalmente por adultos jovens do sexo masculino, vítimas de acidentes de motocicleta, o que condiz com o perfil demográfico típico relatado de pacientes com LTTPB em todo o mundo. 1 7 10

A recuperação da flexão do cotovelo por meio da neurotização do nervo frênico tem sido demonstrada de forma consistente, o que corrobora nossos achados. Nesta série de casos, observamos níveis de força segundo o MRC até o grau III em 81,3% dos pacientes, semelhante aos 80% relatados recentemente por Hussain et al. 13 e os 70% documentados na única revisão sistemática encontrada sobre o assunto. 9 A recuperação até o grau IV foi observada em 43,8% dos casos, taxa comparável aos 62% relatados por Socolovsky et al. 14 O tempo entre a lesão e a cirurgia em nosso estudo foi, em média, de 7 meses, ligeiramente superior aos 4 a 5 meses relatados em outras séries. Isso sugere que a transferência do nervo frênico pode ser aplicável até mesmo em tratamentos um pouco tardios, como pode ocorrer em serviços públicos e centros de referência. No entanto, há necessidade de cautela, uma vez que cirurgias realizadas nos primeiros 4 meses após a lesão resultaram em recuperação de MRC de grau III em 96% dos casos, ao passo que cirurgias posteriores alcançaram este grau em apenas 43% dos casos. 9

Embora a escala do MRC forneça boas informações sobre a força muscular, medidas quantitativas são cruciais para entender a magnitude da recuperação da flexão do cotovelo. Sokolovsky et al. 8 14 foram, até agora, os únicos autores a relatar de forma objetiva a força de flexão do cotovelo em pacientes com LTTPB, com resultados que variaram de 21 a 29% em relação ao membro saudável, em média, após um período pós-operatório mínimo de 10 meses e médio de cerca de 36 meses. Infelizmente, os autores 8 14 relataram apenas médias, o que impossibilita a avaliação da variabilidade na recuperação da força. No entanto, com base em nossos resultados e na prática clínica, é razoável esperar uma grande variabilidade, decorrente de vários fatores, inclusive relacionados ao paciente (como a extensão da lesão, o tempo entre a lesão e a cirurgia, o sexo ou a idade, por exemplo) e à cirurgia (como a qualidade do enxerto, os procedimentos técnicos ou a experiência da equipe, por exemplo). Embora seja difícil inferir as causas da variabilidade a partir dos dados disponíveis, medidas objetivas oferecem uma avaliação quantitativa da recuperação da flexão do cotovelo, o que contribui para documentar a evolução dos resultados ao longo do tempo e em diferentes abordagens técnicas. Além disso, tais medidas permitem uma compreensão mais abrangente das condições do paciente, uma vez que a obtenção de graus de MRC até IV 9 é comum neste cenário; no entanto, não é realista esperar uma recuperação quase perfeita do controle da flexão do cotovelo nesses pacientes.

Observamos comprometimentos inconsistentes na função respiratória dos pacientes, mas não sintomas respiratórios. Essa ausência de sintomas está de acordo com os achados de estudos anteriores sobre a transferência do nervo frênico. 8 13 15 16 Da mesma forma, a paralisia diafragmática já foi observada sem manifestação clínica. 10 Nosso estudo relatou valores de CVF e VEF1 menores do que o esperado em indivíduos saudáveis, o que reflete uma possível diminuição da função pulmonar, condizente com relatos anteriores de diminuição de 10% no VEF entre antes e 30 meses após a cirurgia. 15 16 Este estudo não dispõe de dados longitudinais para o acompanhamento das alterações individuais na função respiratória ao longo do tempo. No entanto, é razoável antecipar uma tendência de recuperação pós-operatória caso a função respiratória tenha sido de fato acometida. A ausência de tal tendência sugere que a variabilidade observada na função respiratória pode ser influenciada por outros fatores, como a diminuição da atividade física. Estudos em cadáveres 17 18 indicam que um nervo frênico acessório pode estar presente em 48 a 61% dos indivíduos, o que poderia explicar por que metade dos nossos pacientes apresentou CVF normal após a cirurgia. Em suma, as evidências de déficits respiratórios são inconsistentes e não parecem suficientes para causar sintomas respiratórios.

A avaliação do controle muscular é fundamental em pacientes submetidos à transferência neural, pois a função motora original pode diferir da função do músculo reinervado. O nervo frênico apresenta algumas vantagens na reinervação do bíceps braquial, como o fato de compartilhar uma origem embrionária comum com o plexo braquial e ser facilmente acessível e rico em fibras mielinizadas em comparação a outras opções extraplexuais. 19 Porém, a natureza intermitente da ativação diafragmática difere dos padrões esperados de flexão do cotovelo em situações cotidianas, 14 e a ativação involuntária do bíceps braquial pode ser observada em alguns pacientes durante os movimentos respiratórios. 8 Nossos resultados mostram uma tendência temporal para essa ativação involuntária, que se inicia após um período inicial de recuperação, provavelmente devido à cicatrização do enxerto, atinge o pico nos primeiros anos pós-operatórios, e, então, é controlada, o que reflete o fenômeno da neuroplasticidade. Curiosamente, pesquisas anteriores 8 observaram que, em pacientes com recuperação da flexão do cotovelo de nível III na escala do MRC, a inspiração forçada máxima antes da flexão do cotovelo melhora a produção de força em comparação à expiração forçada máxima. Neste estudo, não observamos diferenças relacionadas às manobras respiratórias. Alguns de nossos pacientes apresentaram níveis menores de recuperação da flexão do cotovelo. Assim, é possível que tal interferência seja acentuada em pacientes com recuperação melhor, o que merece atenção em estudos futuros. De modo geral, esses resultados destacam a capacidade do sistema nervoso central de se adaptar à nova configuração do nervo frênico.

Este estudo apresenta algumas limitações dignas de nota. Dinamômetros isocinéticos podem subestimar a força de indivíduos não familiarizados com o equipamento. Adaptamos o dispositivo para não exigir a manipulação da alça, o que facilita seu manuseio por indivíduos com déficits de preensão manual. No entanto, equipamentos desenvolvidos especificamente para avaliação nessa população podem ser mais apropriados. Além disso, a ativação muscular involuntária foi normalizada pela ativação voluntária durante a contração máxima. A normalização pela capacidade máxima de ativação intrínseca do músculo, utilizando ondas M obtidas por eletroestimulação, pode ser considerada em investigações futuras.

## Conclusão

A transferência do nervo frênico restaurou efetivamente a flexão do cotovelo na maioria dos pacientes. Embora as manobras respiratórias não tenham influenciado a produção de força, houve ativação involuntária do bíceps braquial durante os ciclos respiratórios forçados, com pico após um período inicial de recuperação e subsequente declínio. Não encontramos evidências de comprometimento pulmonar grave nesses pacientes, apesar dos sinais de paralisia diafragmática.
